# The Cost-Effectiveness of Different Feeding Patterns Combined with Prompt Treatments for Preventing Mother-to-Child HIV Transmission in South Africa: Estimates from Simulation Modeling

**DOI:** 10.1371/journal.pone.0102872

**Published:** 2014-07-23

**Authors:** Wenhua Yu, Changping Li, Xiaomeng Fu, Zhuang Cui, Xiaoqian Liu, Linlin Fan, Guan Zhang, Jun Ma

**Affiliations:** Department of Health Statistics, College of Public Health, Tianjin Medical University, Tianjin, China; Alberta Provincial Laboratory for Public Health/University of Alberta, Canada

## Abstract

**Objectives:**

Based on the important changes in South Africa since 2009 and the Antiretroviral Treatment Guideline 2013 recommendations, we explored the cost-effectiveness of different strategy combinations according to the South African HIV-infected mothers' prompt treatments and different feeding patterns.

**Study Design:**

A decision analytic model was applied to simulate cohorts of 10,000 HIV-infected pregnant women to compare the cost-effectiveness of two different HIV strategy combinations: (1) Women were tested and treated promptly at any time during pregnancy (Promptly treated cohort). (2) Women did not get testing or treatment until after delivery and appropriate standard treatments were offered as a remedy (Remedy cohort). Replacement feeding or exclusive breastfeeding was assigned in both strategies. Outcome measures included the number of infant HIV cases averted, the cost per infant HIV case averted, and the cost per life year(LY) saved from the interventions. One-way and multivariate sensitivity analyses were performed to estimate the uncertainty ranges of all outcomes.

**Results:**

The remedy strategy does not particularly cost-effective. Compared with the untreated baseline cohort which leads to 1127 infected infants, 698 (61.93%) and 110 (9.76%) of pediatric HIV cases are averted in the promptly treated cohort and remedy cohort respectively, with incremental cost-effectiveness of $68.51 and $118.33 per LY, respectively. With or without the antenatal testing and treatments, breastfeeding is less cost-effective ($193.26 per LY) than replacement feeding ($134.88 per LY), without considering the impact of willingness to pay.

**Conclusion:**

Compared with the prompt treatments, remedy in labor or during the postnatal period is less cost-effective. Antenatal HIV testing and prompt treatments and avoiding breastfeeding are the best strategies. Although encouraging mothers to practice replacement feeding in South Africa is far from easy and the advantages of breastfeeding can not be ignored, we still suggest choosing replacement feeding as far as possible.

## Introduction

Identification of human immunodeficiency virus (HIV) infection is critical from both clinical and public health perspectives. Antenatal HIV testing is undertaken primarily to offer interventions to reduce the risk of HIV transmission from mother to child. Globally, about 40% of pregnant women in low- and middle-income countries received HIV testing and counseling in 2012, up from 26% in 2009 [1].These prevention of Mother-to-child transmission (PMTCT) services in low- and middle-income countries have prevented approximately 409,000 children from acquiring HIV [2]. Around 330,000 children were HIV-infected prenatally in 2011, which represented a decline of 24% since 2009 in sub-Saharan Africa [2]. Especially the implementation of South Africa's massive HIV testing and counseling campaign between April 2010 and June 2011,which urged everyone in 12–60 years old to be tested [3], caused the national testing coverage to exceed 95% in 2012 [2].

However, there are the persons who are tested late or are unaware of their infection until relatively late in their disease course, as a result, missing the opportunity of getting prompt intervention. A World Health Organization (WHO) study of HIV diagnoses in Georgia in 2009–2011 found that 64% of new HIV diagnoses could be considered ‘late’ and that the reasons for the high rates of late diagnosis included lack of access to acceptable HIV testing and counseling services [2]. An interview study of 760 HIV-infected persons in Los Angeles County suggested that many persons reported with Acquired Immune Deficiency Syndrome (AIDS) were unaware of their infection until relatively late in their disease course. Of particular concern is that almost half (46%) of the reported respondents did not seek testing until they were ill [4]. A study based in two Durban clinics found most patients were tested at a late stage of infection with over 60% of CD4 counts below 200 cells/mm^3^.Of those who were eligible for treatment, more than a fifth died, mostly before any treatment [5]. A survey had suggested that late HIV diagnosis may lead to accelerted progression and that some of the patients in the survey developed AIDS within a year of HIV infection[6]. These findings are in excellent agreement with those reported by other researchers [7].

Since the first report of transmission of HIV through breastfeeding was published in 1985 [8], avoidance of breastfeeding has remained an important component of efforts to prevent mother-to-child transmission (MTCT) of HIV [9]. There is no doubt that breastfeeding is risky for MTCT [8], [10] and approximately 5%–20% of babies infected through MTCT acquire HIV infection via breastfeeding [2], but breastfeeding may be associated with other factors. Breastfeeding is particularly important in resource-poor regions of the world, where limited access to clean water increases the risk of diarrhea if replacement feeding is used, and many mothers do not have the means to afford the cost of formula [11]. Infant morbidity and mortality rates are generally decreased by breastfeeding, which provides optimal nutrition and partially protects against common childhood infections [11]–[13], [20].

On account of realistic public health considerations, in 2010, WHO issued its first guidelines [14] that allowed new recommendations on antiretroviral (ARV) prophylaxis to either the mother or infant during breastfeeding in areas where breastfeeding was judged to be the most appropriate choice of infant feeding for HIV-infected women. In addition, guidelines were developed to provide international standards to reduce the risk of MTCT from a background risk of 35% to less than 5% (or even lower) in breastfeeding populations [14]. Exclusive breastfeeding for the first few months of life can be successfully supported in HIV-infected women [2] and if replacement feeding is not available [15], one alternative is to provide antiretroviral prophylaxis (ART) to the mother or child during breastfeeding [16]–[19].

HIV/AIDS in South Africa is a prominent health concern [21]. The Joint United Nations Programme on HIV and AIDS (UNAIDS) report estimated that 5,700,000 South Africans had HIV/AIDS, with HIV prevalence in pregnant women at 28% [22], or just under 12% of South Africa's population of 48 million in 2007 [23]. However, important changes have occurred in the country since 2009 [24]. Government funding in South Africa increased for expansion of antiretroviral therapy, scaling up of PMTCT programmes, promotion of HIV and tuberculosis treatment integration, and increased investments in HIV prevention [25]. South Africa now has the world's largest programme of antiretroviral therapy, with about 1.8 million people estimated to be taking antiretroviral. HIV prevention has received increasing attention [24].

Breastfeeding is the norm in South Africa, but the percentage of exclusive breastfeeding for 6 months among South African HIV infected mothers is one of the lowest in the world, at 8% in 2003 [26], [27]. However, the situations have been changed since 2010 when the infants feeding patterns shifted the emphasis to exclusive breastfeeding [24], and as a result, South Africa has had one of the sharpest declines in new infections among children [28]. Yearly infections in children have dropped from 56,500 in 2009 to 29,100 in 2011. [29] A cohort study of 1032 HIV-infected mothers showed that 40% of women reported to exclusive breastfeeding [30].There were approximately 45% of HIV mothers reported as exclusive breastfeeding in another Kesho Bora study [16].

It should be noted that the cost of drugs to HIV are usually borne by the government, whereas formula is usually paid for by the individual. Meanwhile, encouraging HIV mothers to practice exclusive breastfeeding is also far from easy. Breast milk provides all of the fluids and nutrients that a young baby requires, so it means that even water should be avoided [31], [32]. However, in many societies, it is normal for a baby to be given water, tea, porridge or other foods as well as breast milk, even during the first few weeks of life [33], [34]. The HIV-infected mothers assigned to the formula feeding often experience community, family, or spousal pressure to breastfeed and are sometimes concerned about maintaining the confidentiality of their HIV status; further, formula-feeding logistics are more difficult than those for breastfeeding, particularly in resource-poor areas [14].

Numerous simulations [35], [36] have been conducted to prevent MTCT. However, most of them explored the effect of only a single intervention. For example, some studies evaluated the cost -effectiveness of antenatal HIV testing but the remedial measures for untested or testing late HIV-infected mothers were not included [37]–[40]. Neil Soderlund et al. simulated the cost-effectiveness of four feeding strategies and three antiretroviral interventions but the interventions were considered separately [40]. In practice, however, interventions should not be implemented individually and several interventions can be implemented simultaneously or consecutively. Based on the important changes in South Africa [24] and the South African Antiretroviral Treatment Guideline 2013 recommendations [26], we explored different strategy combinations rather than a single intervention according to the South African HIV-infected mothers' varying status and different designs of feeding patterns. We simulated the different status(prompt treatment, remedial treatments or neither) of HIV infected mothers based on the characters of South African pregnant women in Kesho Bora study and others[16], [30], [41], [42], and we aimed to assess the cost-effectiveness of the feeding patterns of HIV-exposed infants (exclusive breastfeeding or replacement feeding) in light of the mothers' status.

## Materials and Methods

### Model framework

**Figure 1:**
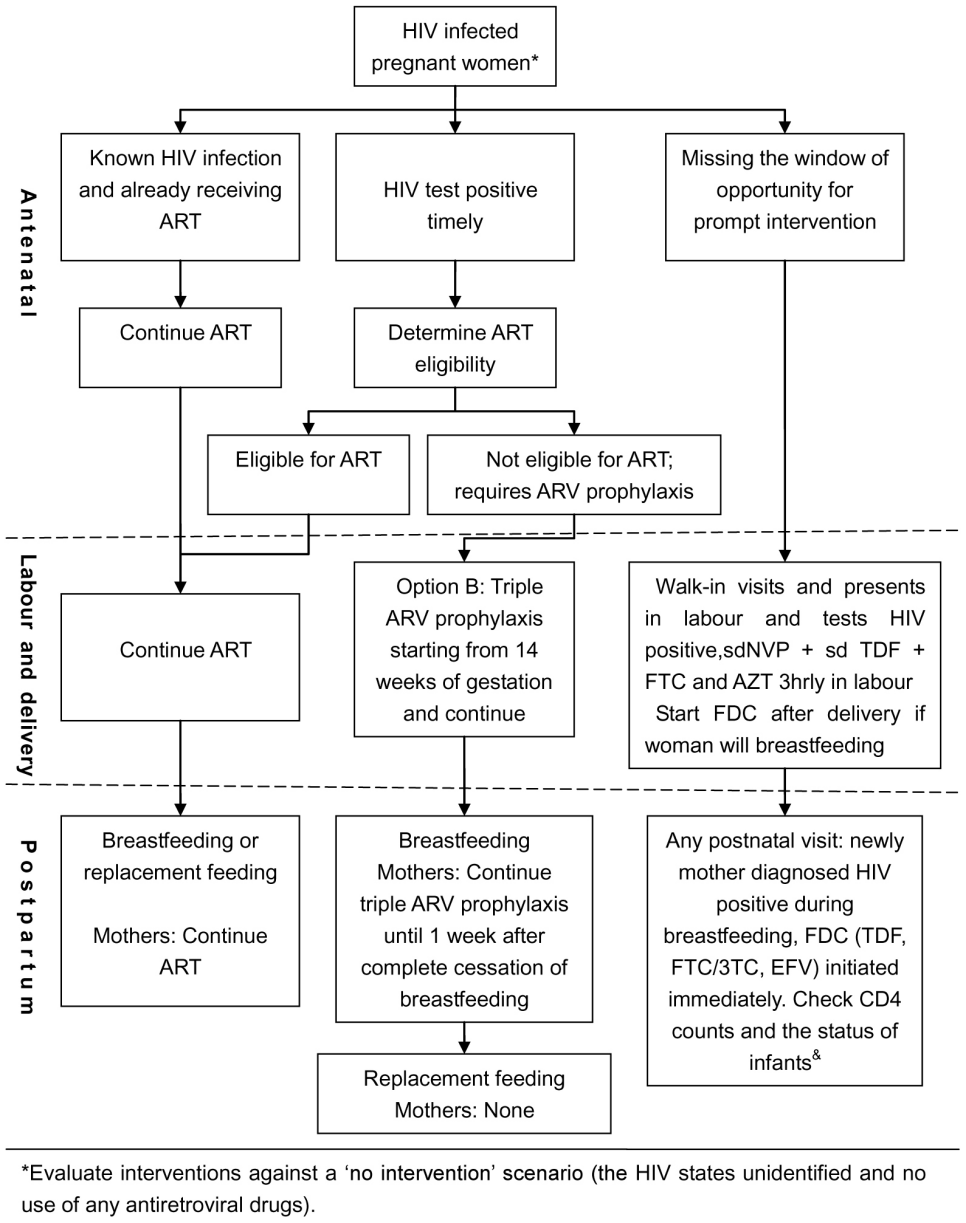
Decision analytic model schematic. & Irrespective of mode of feeding patterns, infants accepted NVP promptly and daily for 6 weeks. ART, antiretroviral therapy; ARV, antiretroviral prophylaxis; sdNVP, single dose nevirapine; sdTDF, single dose Tenofovir; FTC,emtricitabine; AZT, Zidovudine; FDC, fixed dose combination,(TDF, FTC/3TC, EFV); 3TC,Lamivudine; EFV, Efavirenz.

**Figure 2:**
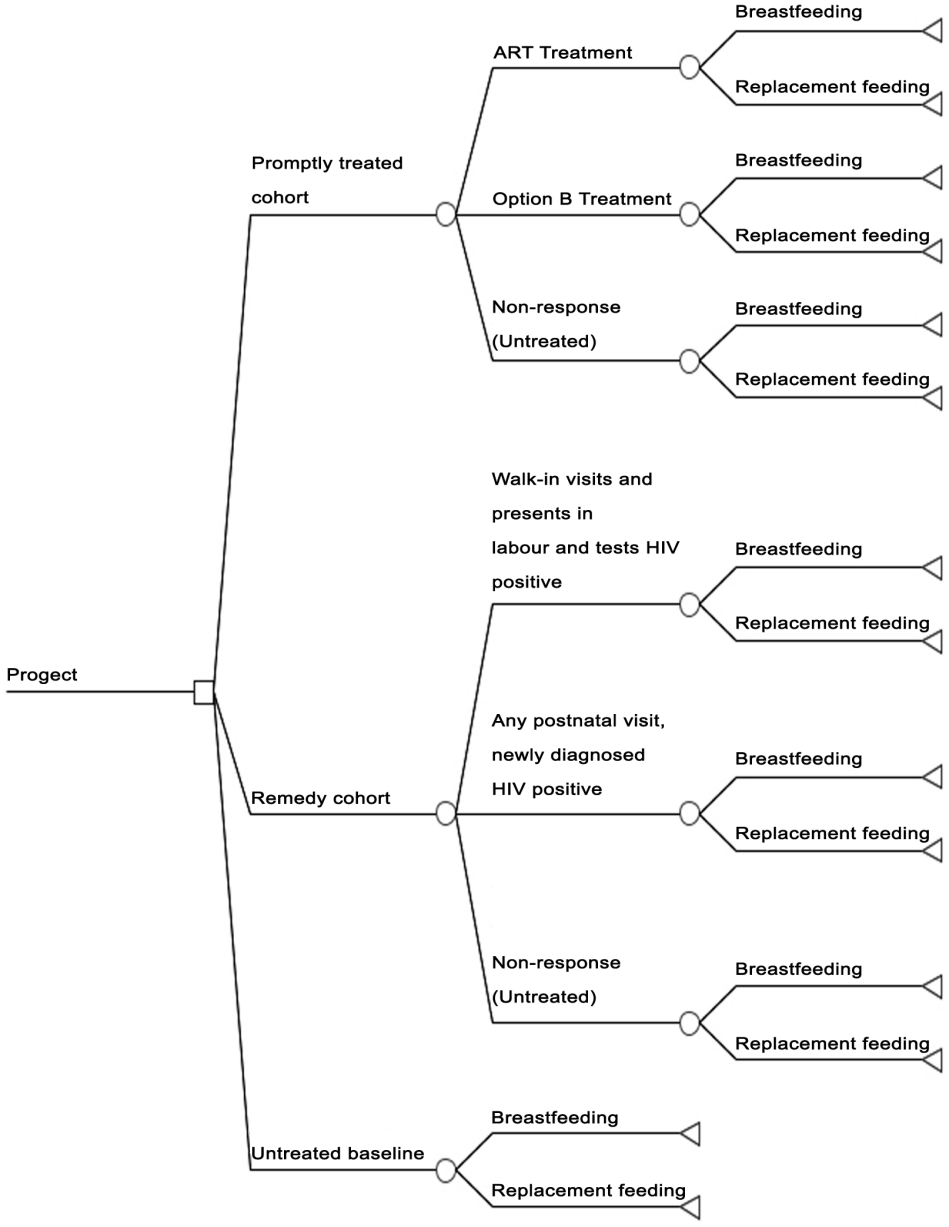
Structure of decision analytic model. ART, antiretroviral therapy.

We developed a decision analytic model using the TreeAge Pro 2011 software package (TreeAge Software, Inc, Williamstown, MA) [45] to compare the cost-effectiveness of two different HIV strategy combinations given only to HIV positive mothers: (1) HIV positive pregnant mothers were tested and treated promptly at any time during pregnancy. They knew their HIV status after antenatal HIV testing in time and received an ARV intervention or standard ART recommended by WHO on the basis of eligibility (Promptly treated cohort). (2) Pregnant mothers did not get tested or treated until after delivery. They presented late in labor without having a diagnosis of their HIV status and thus missed the window of opportunity for prompt interventions. A course of fixed dose combination (FDC) and other treatments were offered as a remedy (Remedy cohort). The first strategy was the gold standard, whereas the second one was remedial when the first wasn't available. The non-responders were assigned as untreated in the two cohorts, and replacement feeding or exclusive breastfeeding was assigned in the two strategies. We evaluated interventions against a ‘no intervention’ scenario (the mothers' HIV status unidentified and no use of any antiretroviral drugs). The model structures are showed in Figure 1 and Figure 2.

We presented key model outcomes, including the number of infant HIV cases averted, the number of infant life years (LY) saved, the cost per infant HIV case averted, and the cost per LY [40], [45] saved from the interventions.

### Intervention and settings

The decision analytic model was created to simulate 3–6 cohorts. We used the Monte Carlo simulation method to simulate 10,000 HIV-infected pregnant women from South Africa (average age is 27 years old) in each cohort [16]. The distribution of the pregnant mothers' age, the CD4 counts and other parameters were all based on the characters of South African pregnant women in Kesho Bora study and others [16], [30], [41], [42]. We simulated the severity and progression of the maternal HIV disease, infants' HIV infections and the time that mothers' highly active antiretroviral therapy (HAART) treatments started based on mothers' CD4 counts during pregnancy. The median CD4 cell count was assumed to be 360×10^6^ cells/mm^3^. The square root of CD4 count is assumed to be normally distributed within a 95th percentile range of 43 to 984 cells/mm^3^ [37]. We assumed that HIV-infected, pregnant women who have CD4 cell counts less than 350 cells/mm^3^ received HAART during pregnancy, as the WHO recently recommended [14]. The intervention approaches assumed that maternal antepartum daily ART continued during pregnancy, delivery, and thereafter. For women who were not eligible for ART, the model used implementation of triple antiretroviral prophylaxis (Zidovudine (AZT) + Lamivudine (3TC) + lopinavir (LPV)) [14], as did the Kesho Bora study conducted in South Africa [16]. Triple ARV prophylaxis started as early as 14 weeks of gestation and continued until delivery; if breastfeeding was applied, it continued until cessation of breastfeeding at 6 months postpartum. All the infants received daily nevirapine (NVP) from birth until age 6 weeks, regardless of the mode of infant feeding.

For HIV-infected mothers who missed the window of opportunity for prompt intervention, some of them were unable to realize their HIV infectious status, which resulted in walk-in labour visits and delayed HIV testing; single-dose nevirapine (sdNVP) + single dose Tenofovir (sdTDF) + emtricitabine (FTC) + Zidovudine (AZT) were then offered at the onset of labor as a remedy, and NVP was also given to their newborn babies daily for 6 weeks [26]. A course of fixed dose combination (FDC) was started after delivery if the woman was assigned to breastfeeding. For others who were diagnosed HIV-positive during breastfeeding at any postnatal visit, FDC (TDF, FTC/3TC, Efavirenz (EFV)) was initiated immediately, and the mothers' CD4 counts and their infants' HIV status were checked. All the interventions mentioned above were in line with recommendations by the South African Antiretroviral Treatment Guideline 2013 [26]. We identified these detailed interventions to ensure the accuracy of the intervention cost and effect.

We also assumed a standard testing strategy consisting of a serum enzyme-linked immunosorbent assay followed by confirmatory Western blotting. We presumed infants complied with exclusive breastfeeding from ages of about 6 weeks to 6 months if their mothers intended to breastfeed, as the WHO recommended [44].

### Probabilities

The probabilities and ranges used in the model were derived from published studies of large, population-randomized, controlled trials and published, updated meta-analyses [11], [12], [15], [16], [41], [43]. We obtained estimates for the model's parameters from South African data when available. Otherwise, we used data from international literatures in an attempt to use estimates from other resource-limited countries. A function to convert annual probability into 18-month probability was used, as follows:
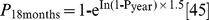



The base rates of MTCT were estimated from a randomized controlled trial conducted in Kenya and South Africa by the Kesho Bora study group [16]. In a group of those eligible for ART, the average HIV-positive rate in infants who were breastfed for 18 months was 7.5% (compared with 0.75% in the replacement-feeding group). A range of 3.5%–14.76% was included in the sensitivity analyses, taking the impact of CD4 counts into account. In the group of mothers whose CD4 counts were ≥350 cells/mm^3^ and who were assigned to breastfeeding, the converted 18-month infection rate was 6.97% (95% confidence interval (CI) 3.58%–13.62%). The sensitivity analysis includes a lower transmission rate of 1%, derived from an 18-month follow-up study of HIV–infected mothers whose CD4 counts were ≥500 cells/mm^3^ in the Kesho Bora study [16], [41]. The risk of MTCT was 2% with the use of antiretroviral medicines and avoidance of breastfeeding [43]. 19.5% of exclusively breastfed infants were infected with HIV by 6 months, a figure derived from a intervention cohort study of 1132 HIV positive pregnant women [30]. We used the vertical MTCT rate of HIV infection during postpartum of 15.81% (95% CI 10.7%–21.02%) and 29.03% (95% CI 22.98%–35.26%) without interventions to prevent transmission when the mother was assigned to breastfeeding or replacement feeding, respectively [13], [78].

On average, 59% (95% CI 53%–64%) of pregnant women living with HIV were estimated to be eligible for ART on the basis of those with CD4 counts <350 cells/mm^3^ receiving lifelong ART [1]. The 83% and 67% South African coverage rates of ART among adults and children (aged 0–14 years), respectively, were assumptions made on the basis of the WHO's Global Update on HIV Treatment 2013 Report [1]. The latest data from 23 countries indicated that the average retention rates for people on ART decrease over time (from about 86% at 12 months to 82% at 24 months) [1]. We assigned 40% and 15.4% as the percentage of women who were selected to exclusive breastfeeding and replacement feeding respectively [30]. Since data to measure precisely the efficacy of remedial intervention in labor and that of FDC during postpartum care are not available, we estimated the variable efficacy of the remedial intervention in labor as 62.75% (95% CI 40.76%–84.74%), derived from data of the Petra study [46], [47]. We rounded the efficacy of FDC to 47.91% (95% CI 43.57%–51.92%), this figure came from a research of 2,127 electronic medical records between July 1999 to June 2006.The hazard ratio (HR) of FDC was 0.92 [48]. In addition, we assumed that the converted 18-month mortality in exclusive breastfeeding infants was 18.9% versus 15.4% in infants given replacement feeds [49], and calculated the adjusted mortality rate which represents the unrelated HIV infection mortality by deducting the under-five mortality rate (42.15/1000 in 2013) [50]. The input probabilities for our decision tree are displayed in Table 1.

**Table 1:** References and input probabilities for the decision analytic model.

Reference	Probability Variable	Details	Circumstances	Value	Range
[16]	positive rate of HIV in 18 months	Breastfeeding	ART	7.50%	(3.50%–14.76%)
[16] [32]			ARV	6.97%	(1.00%–13.62%)
[13] [11]			None treatment	29.03%	(22.98%–35.26%)
[43]		Replacement feeding	ART	0.75%	(0.75%–1.50%)
[43]			ARV	2.00%	-
[13] [78]			None treatment	15.81%	(10.70%–21.02%)
[30]	Efficacy	Using sdNVP + sdTDF + FTCand AZT 3hrly	In labour as a remedy	62.75%	(40.76%–84.74%)
[48]		Initiating FDC immediately	Breastfeeding during postpartum care	47.91%	(43.57%–51.92%)
[2]	Rate of coverage	ART	Among pregnant women	83.00%	(79.00%–87.00%)
		ART	Among infected infants	67.00%	(60.00%–75.00%)
[2]		HIV testing and counseling	Among pregnant women	95.00%	(90.00%–98.00%)
Assumed, [16] [27] [30]		Exclusive breast feeding	In HIV infected women	40.00%	(8.00%–44.68%)
[30]		Replacement feeding	In HIV infected women	15.4%	-
					
[2]	Rates of average retention	ART	In 12 months	86.00%	-
			In 24 months	82.00%	-
[13]	Estimated rate of transmission	Breast milk		19.50%	(6.50%–24.90%)
[2]	Average rate of being eligible for ART	Based on CD4<350	Mothers living with HIV	59.00%	(53.00%–64.00%)
Assumed [60],	Proportion of HIV	Infected women among the unidentified	Presented in labor	18.52%	(5.00%–50.00%)
			Diagnosed at postnatal visit	22.22%	(5.00%–50.00%)
[24]	Life experience(year)	Child	Without HIV	60	-
[53] [54]			With prenatal HIV on HAART	40	-
[55]			With prenatal HIV if no no antiretroviral	10	-
[42] [43]			With AIDS	1	-
[50]		Under-five mortality rate		42.15‰	-
[49]	Motility rate of infants in 18 months	Breastfeed		18.9%	-
[49]		Replacement feeding		15.4%	-

ART, antiretroviral therapy; ARV, antiretroviral prophylaxis; sdNVP, single dose nevirapine; sdTDF, single dose Tenofovir; FTC, emtricitabine; AZT, Zidovudine; FDC, (TDF, FTC/3TC, EFV); 3TC,Lamivudine; EFV, Efavirenz; HAART, highly active antiretroviral therapy.

### Cost estimates

The input cost estimates for our decision tree are presented in Table 2. Cost and utility estimates were also derived from published literatures when necessary. All cost was expressed in 2012 US dollars and South African prices. The total cost included the cost of ART and ARV treatments, the cost of visiting, counseling and testing and the cost of breastfeeding and formula milk [58]. We calculated the cost of mothers from the moment when treatments begin until 1 week after cessation of breastfeeding and the cost during the first 18 months of life for the HIV-exposed infants. The cost-effectiveness was calculated relative to the control group as (IC+NC−HC)/(LS*LE−LL*LE) [40]; where IC  =  intervention costs, NC = health care costs because of additional morbidity in formula fed children not infected with HIV, HC = costs of HIV related care avoided by preventing infections, LS = lives saved by prevention of HIV infection, LL = lives lost because of formula feeding in children not infected with HIV, and LE = life expectancy. Cost estimates for antiretroviral drugs were based on the Clinton Health Access Initiative (CHAI)'s ceiling price list [51]. The average of the two most common first-line regimens (zidovudine/lamivudine/nevirapine or tenofovir/lamivudine/efavirenz, $146.50/year) were used as the cost of triple ARV during pregnancy [42]. The cost of HAART during pregnancy and lactation were estimated as $76.82 and $50.65, respectively [53], [79]. We estimated the baseline cost of a single rapid HIV testing as $2.36 and $6.30, when the testing was negative or positive, respectively [53]. In case of a positive testing, additional confirmatory testing was needed. The cost of CD4 testing was estimated as $5.43 per unit [43], [52]. Cost ware discounted by 3% per year [45].

**Table 2:** References and input cost estimates for the decision analytic model.

Reference	Composition of cost	Items	Value($)(year = 2012)
[43] [52]	Testing cost	CD4 testing	5.43
[53]		ELISA testing	2.10
[53]		Positive rapid HIV testing	2.36
[53]		ELISA testing and Weston blotting	6.30
[53] [79]	HAART cost	HAART in pregnancy	76.82
[53] [79]		HAART during lactation	50.65
[2]		ART (first-line regimens)	186.00
[43]	ARV cost	Triple ARV in pregnancy(first-line regimens)	146.50
[51]		sdNVP+sdTDF+FTC and AZT in labor per unit	0.44
[51] [43]		FDC(TDF+FTC/3TC+EFV) per year	159.00
[58]	Counseling and Health care cost	Cost of behavior counseling	3.74
[58]	Feeding cost	Breastfeeding (6 month)	153.98
[58]		Formula feeding (6 month)	310.82

ELISA, enzyme-linked immunosorbent assay; HAART, highly active antiretroviral therapy; ART, antiretroviral prophylaxis; ARV, antiretroviral prophylaxis; sdNVP, single dose nevirapine; sdTDF, single dose Tenofovir; FTC, emtricitabine; AZT, Zidovudine; FDC, (TDF, FTC/3TC, EFV); 3TC,Lamivudine; EFV, Efavirenz.

### Estimation of life years saved

We focused our analysis on the effectiveness for HIV-exposed infants and estimated the effect using the life years saved. To estimate the number of LY saved induced by the intervention, life expectancy in South Africa at birth was estimated from a review of available demographic data [24], and the base-case calculation used a life expectancy of 60 years. For a child with perinatal transmission of HIV who goes on HAART for life, we estimated a life expectancy approximately two-thirds that of a child without HIV [53], [54]. The model used a disease progression scenario in which 25%, 80%, and 100% of children progress to AIDS at 12, 60, and 120 months, respectively. Children were assumed to live for an average of 12 months after progression to AIDS [55], [56].

We used the same weights for all infants, irrespective of age. When children were aged less than 5 years, irrespective of their CD4 counts, we assumed they were eligible to start ART. Children aged greater than 5 years were monitored for ART by CD4 testing each year [26]. The parameters are presented in Table 1.

### Sensitivity and uncertainty analyses

In the sensitivity analyses, the uncertain assumptions about the input cost and behavioral impacts of interventions were varied. An aggregate model was developed using the highest and lowest values and the best fitting parameter. The model's epidemiological values were point estimates and 95% CI for parameters based on published study results. We conducted additional univariate sensitivity analyses with values of cost estimate from one-third to 3 times of their baseline estimates. We also tested the sensitivity of the rankings to variation of the assumptions regarding key parameters.

## Results

**Figure 3:**
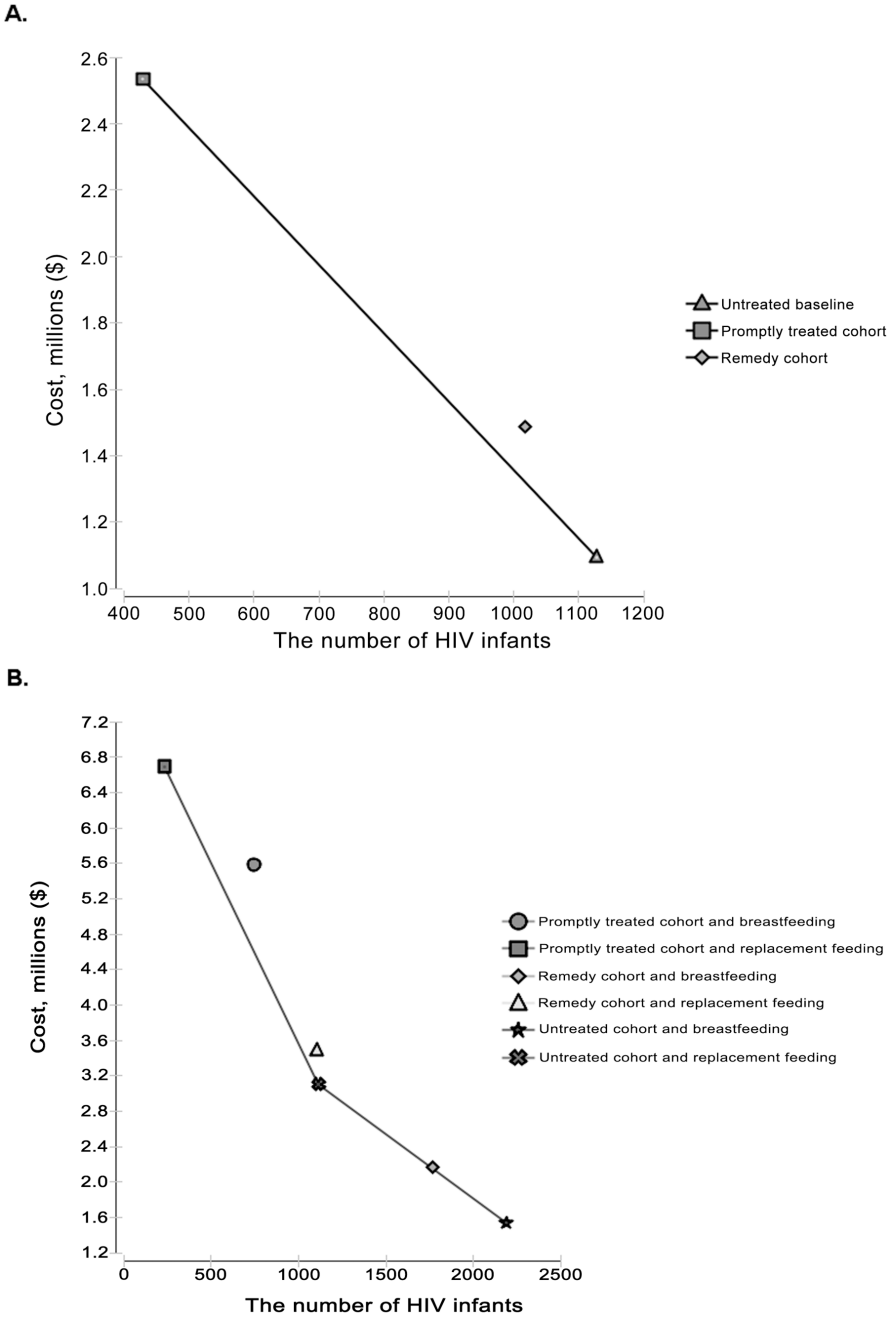
The cost-effectiveness frontier of different strategy combinations. The cost-effectiveness frontier (solid line) includes strategies that maybe cost-effective if the incremental cost-effectiveness ratio is less than the accepted threshold. Strategies that are not on the frontier are dominated, meaning that they are not efficient use of resources. In figure 3.A, irrespective of the feeding patterns, remedial cohort is less cost-effective. In figure 3.B, mothers' prompt treatment and replacement feeding cohort is the most cost-effective intervention, followed by the promptly treated cohort being assigned to breastfeeding.

Three theoretical cohorts(the promptly treated cohort, the remedy cohort and the no intervention control cohort) were applied in our analytic model, all of which were based on estimates of 40% breast feeding coverage and 15.4% replacement feeding rate during postpartum. It is found that the incremental cost per infant HIV case averted were $2063.05 and $3579.66 for the promptly treated cohort and remedy cohort, respectively. Tables 3 reported on the incremental cost-effectiveness ratios (ICER) for interventions, which were listed in the descending order of the infant HIV cases averted. Figure 3 showed an expansion path graphically, with the slope of the line joining any two points indicating the ICER for the more costly option [57]. The remedy strategy did not particularly cost-effective. Compared with the ‘no intervention’ scenario which leaded to 1127 infected infants, 698 (61.93%) and 110 (9.76%) of pediatric HIV cases were averted in the promptly treated cohort and remedy cohort respectively, with incremental cost-effectiveness of $68.51 and $118.33 per LY, respectively (Figure 3.A).

**Table 3:** Results and Outcomes of each cohorts of 10,000 HIV infected pregnant women.

Status	Feeding patterns	Incremental cost US($)	Infant HIV cases averted		Life years saved		Incremental cost-effectiveness
			***Total***	***Incremental cost (per, $)***	***Total***	***Incremental cost(per, $)***	
Baseline	-	-	-	-	-	-	-
(no intervention)							
Promptly treated cohort	-	2063.05	698	1439391.2	21009.8	68.51	Undominated§
Remedy cohort	-	3579.66	110	391806.75	3311	118.33	Extended dominated&
Untreated cohort	Breastfeed	-	-	-	-	-	-
Untreated cohort	Replacement feed	1461.19	1073	1568400	32297.3	48.56	Undominated
Promptly treated cohort	Replacement feed	4059.88	883	3584863.5	26578.3	134.88	Undominated
Remedy cohort	Breastfeed	1508.69	421	635167.86	12672.1	50.12	Extended dominated
Promptly treated cohort	Breastfeed	5826.72	360	2094217.3	10836	193.26	Extended dominated
Remedy cohort	Replacement feed	37296.63	11	383788.86	331.1	1159.13	Extended dominated

& : extended dominated, means exclude any interventions that have a higher ICER than more effective interventions.

§ : undominated, strategies on the cost-effectiveness frontier, meaning that they are more cost-effective.

Because all the HIV-exposed mothers among the two cohorts were designed to have different feeding patterns, we also conducted a subgroup analysis according to the feeding patterns, using the untreated cohort (Untreated mothers who were assigned to breastfeeding) for comparison. Compared with the untreated cohort, assigning promptly treated cohort to the replacement feeding strategy was the more cost-effective ($134.88 per infant LY saved), followed by the promptly treated cohort being assigned to breastfeeding ($193.26 infant LY saved), as showed in Figure 3.B. With or without the antenatal testing and treatments, breastfeeding was less cost-effective than replacement feeding strategies.

### Sensitivity Analysis

Since the actual values can vary in different settings, both one-way and multivariate sensitivity analyses were conducted to examine the factors that account for the variation in the cost per LY saved. The promptly treated cohort retained a cost-effectiveness ratio lower than $120 per LY in all one-variable sensitivity analyses. When we varied our uncertain assumptions regarding the input parameters, the ranking of interventions remained stable.

If the treatment efficacy of FDC ranged from 25% to 75%, the cost per LY would range $76.5–$223.8 in the remedy cohort. Although the ranking of interventions remained stable under these assumptions, the treatment efficacy of FDC in postnatal intervention significantly influenced the results.

When we decreased the rate of breastfeeding coverage in HIV-infected women from 100% to 5%, the incremental cost-effective per LY increased from $39.02 to $52.95 and from $67.97 to $406.94 in the prompt treated cohort and remedy cohort, respectively. Breastfeeding had a stronger influence on the remedy cohort than the promptly treated cohort. When the rate of breastfeeding coverage in HIV-infected women was reduced by 5% each time, about 57 and 19 infants might be avoided in the promptly treated cohort and remedy cohort, respectively.

Variation in the proportion of newly HIV-infected women presenting in labor and at postnatal visits also substantially affected the result of the remedy cohort. The proportion of HIV-infected women presenting in labor was set to vary by up to 3 times of the point estimate which could entail a ratio as high as 32% of the point estimate ($157 per LY) in the remedy cohort. Similarly, when the proportion of HIV-infected women treated during the postnatal visits increased from the base-case value of 22% to 80%, the cost per LY increased from $59.20 to $95.15 per LY in the remedy cohort. In conclusion, without consideration of willingness to pay, remedial treatments both in labor and at postnatal visits were worthwhile.

## Discussion

In this study, we developed a decision analytic model for prevention of mother-to-child HIV transmission and applied it to discuss the cost-effectiveness of antenatal HIV testing and treatments recommended by WHO versus the interventions used as a remedy in labor and the postpartum period after missing the window of opportunity for prompt intervention.

Early knowledge of HIV status may enable women to make more appropriate decisions with regard to their own health and that of their unborn children [59]. Our study reveals that antenatal testing and prompt treatment will prevent more than half of pediatric HIV cases. Knowledge of the mother's infection status can save an additional 5.9 pediatric LY [37]. There was a correlation coefficient of .66 between the period of confirmed HIV status and receipt of ARV, which illustrated that later confirmation of HIV-infected status led to less possibility of receiving ARV among mothers; this conclusion was derived from an analysis of 108 HIV-infected mothers in China [60]. One research in Hong Kong is also in support of this point of view [61].

Attempts to compare the cost and benefits for testing late and untested HIV-infected women have been previously undertaken [37]–[39]. However, these analyses failed to account for receipt of remedies in labor and during the postnatal visits among untested HIV-infected women. The guideline in South Africa recommends all pregnant women who need triple therapy and breastfeeding to receive a FDC compatible regimen as a remedy [26], and FDC has been rolled out in South Africa since 2013 [62]. We found that those remedial treatments would avert 9.76% of pediatric HIV cases compared with lack of treatment.

Furthermore, since the differences between the promptly treated cohort and remedy cohort are obvious, our focus was to study the impacts of different feeding patterns and breastfeeding coverage between the two cohorts.

Our model is in good agreement with other studies [12], [35], [60] and demonstrates that formula feeding results in a substantial decrease in HIV transmission risk and is cost-effectiveness, but formula is sometimes unaffordable for most HIV infected women because of the resource-limited settings[14], [40]. As our results show, although breastfeeding strategy is less cost-effective, we can't ignore the impact of willingness to pay. In sub-Saharan Africa, more deaths would be caused than saved by formula feeding [40]. Breast milk contains nutrients, agents and antibodies that protect the infant from the risk of childhood diseases such as diarrhoea. Without being breastfed, an infant runs the risk of becoming seriously ill with diseases other than HIV. Where treatment for them is limited or inaccessible, an infant's health can be compromised. Similarly, unsafe and unreliable replacement feeding when clean water and resources are unavailable can also be a danger to an infant's health [64]. Breastfeeding is therefore highly widespread in low- and middle-income countries.

One influential factor for MTCT is the coverage of exclusive breastfeeding. Coovadia et al. confirmed that exclusive breastfeeding from about ages of 6 weeks to 6 months carried an HIV transmission risk of about 4% in South Africa [63]. In our study, each time the rate of breastfeeding coverage in HIV-infected women reduced 5%, there were about 59 and 19 infants whose infections were prevented in the promptly treated cohort and remedy cohorts, respectively. In addition, severity of maternal HIV condition influences the breastfeeding coverage and infants' HIV status. Eighteen-month follow-up of an observational cohort in the Kesho Bora study [41] showed that mothers in less severe disease were more willing to breastfeed their children than mothers in serious condition. Maybe they felt too weak to breastfeed and they were more concerned about HIV transmission or ARV toxicity for their breastfed children. Other researches showed that mothers who chose to replacement feed were more likely to have CD4 counts less than 200 cells/mm^3^ than those who chose exclusively breastfeeding. [30]This opinion was also supported in a South African study [65]. A reference showed a significant correlation between associated mothers'CD4 counts and infants' HIV status. they found that if the concentration of CD4 counts≥350 cells/mm^3^, the HR of infants infected HIV was 0.59 [80]. Delicio found that if the CD4 cell counts lower than 350 cells/mm^3^, it would increase the risk of MTCT more than 12 times. [81]What's more, there was no relationship between gestational age and CD4 counts [82].

Early initiation of ART is important for achievement of an undetectable viral load well before delivery. Thus, women should be encouraged to plan pregnancies and attend antenatal care sufficiently early to diagnose HIV infection, assess the HIV stage, and initiate ART or antiretroviral prophylaxis promptly [16]. The latest WHO guidelines stated that lifelong ARV treatment should be provided for all pregnant women and breastfeeding women with HIV, known as Option B Plus [66]. Countries that do not have the resources to provide lifelong ARV should offer the mother ARV for her own health when she finishes breastfeeding, known as Option B. If the mother is not eligible, she may stop taking ARV one full week after cessation of breastfeeding in low- and middle-income countries. In the 22 priority countries of the Global Plan, there are more than half of them implementing the OPTION B regimen policy for preventing the mother-to-child transmission of HIV among pregnant women living with HIV in 2013 [2]. For example, in South Africa, OPTION B is the main regimen policy because of limited resources [2]. Although providing antiretroviral treatment until complete cessation of breastfeeding (Option B) has numerous advantages and has been preferable in our model, and despite the fact that South Africa recently announce a switch to Option B [63], the switch to Option B will present further challenges, such as operational issues, the cost of increasing the number of women on ART, adherence to lifelong ART, emergence of ARV resistance, and long-term side effects to the fetuses, infants, and mothers [67].

Several limitations of this study deserve mention. First, similar to any cost-effectiveness analysis [53], [68], [69], our study is limited in its inability to model perfectly the complexities of clinical medicine and accurately estimate probabilities and cost. Problems include the lack of empirical data on the effects of FDC [70]–[72] during postnatal intervention. We have used counseling cost but the cost of home visits, Non-Governmental Organization and community involvement was not included in our model. Further, the sensitivity and specificity of each HIV test were not incorporated into the decision tree.

Second, our model does not reflect the practices of elective cesarean section (ECS). ECS before labor has been introduced as an intervention for the PMTCT of HIV and significantly lowers the risk of mother-to-child transmission of HIV infection [73].However, most of the studies of ECS among HIV infected women were conducted exclusively in North America and Europe [74]. In developing countries, such as South Africa, the risks and benefits associated with ECS are seldom explored [74]. The European Mode of Delivery Collaboration Organization had found that the role of mode of delivery in the management of HIV infected women should be assessed in light of risks as well as benefits and the risk/benefits ratio depended upon the underlying rate of MTCT [74].

The ECS rate is lower in South Africa than that of some developed countries, such as 50.7% in France [75]. A multi-country study in Sub-Saharan Africa showed 1276 women underwent ECS, giving a frequency of 6.2% (range 4.1–16.8%) [74]. Further confirmation was also given by another two studies in South Africa, which showed that only 13.24% and 10.92% of HIV-infected mothers underwent ECS, respectively [16], [30], [32]. The low rates indicate that ECS may have not served as ubiquitous practices in South Africa. Thus, the effectiveness of ECS in South Africa is also unable to be evaluated due to inadequate parameters in our model.

Third, mixed feeding during the first several months of life which is not included in our model is a influencing factor of HIV transmission [76]. Mixed feeding infants are nearly 11 times (HR 10.87) more likely to acquire infection than the exclusively breastfed children [30]. However, some women in resource-limited settings are malnourished and their breast milk is not sufficient for their infants [77]. As a result, it is difficult to bring the exclusive breastfeeding into force in South Africa [40].

In addition, establishment of an intervention to reduce vertical transmission of HIV may have important secondary benefits [55], such as prevention of horizontal transmission, vigilant follow-up infant care to prevent opportunistic infections and etc [71]. These benefits have not been quantified in developing countries and hence are not included in the model.

It should be also noted that in this study, interventions for mothers and exposed infants were only evaluated from the moment when the treatment of the mother began until the end of first 18 months of life for the HIV-exposed infants, although the effectiveness associated with these interventions has been estimated for HIV-exposed infants. Meanwhile, the assumption that infants complied with exclusive breastfeeding from ages of 6 weeks to 6 months if their mothers intended to breastfeed may overestimate the results of exclusive breastfeeding. So we draw our conclusion carefully and do not extend the results throughout the whole period of breast milk exposure. The effectiveness of long-term interventions is unknown.

Another limitation in our study is that we extrapolated most assumptions from a limited number of relatively small-scale studies; thus, precise and reliable estimates of the effectiveness of large-scale prevention programs are needed.

Finally, our estimates of new HIV infections do not take into account dynamic spread at the population level or differences at risk populations, and many factors can cause variability in both the cost and effects of interventions.

Despite all these limitations, our model truly reflected the progress of MTCT. We assigned the HIV-infected pregnant women in South Africa as our target population and once pregnant, individuals were assigned different strategies (promptly treated or not). The CD4 counts were also taken into account as to reflect the severity of the HIV disease. Most of the confirmed interventions were included in our model in light of mothers' HIV-status and the corresponding diseases progress, such as the ART treatment, the Triple ARV prophylaxis and the feeding patterns. In addition, we took remedial preventions and the impacts of different feeding patterns into account. Our study should assist the governments of developing countries, such as South Africa, on strategic decision making regarding the health resource allocation.

## Conclusions

In summary, our study demonstrates the cost-effectiveness of antenatal HIV testing and treatments, remedy treatments, and their combinations with different feeding patterns. Antenatal HIV testing and standard prompt treatments constitute a cost-effective strategy even in a resource-limited setting like South Africa. Compared with the promptly treated cohort, remedy during labor or the postnatal period is less cost-effective. Although we should pay more attention to the impact of willingness to pay and the advantages of breastfeeding in resource-limited setting can not be ignored, we still suggest choosing replacement feeding as far as possible. Hopefully, these data will enlighten public health policy decisions in South Africa regarding the implementation of remedial treatments and replacement feeding interventions.
